# Risk of Nonalcoholic Fatty Liver Disease Is Associated with Urinary Phthalate Metabolites Levels in Adults with Subclinical Hypothyroidism: Korean National Environmental Health Survey (KoNEHS) 2012–2014

**DOI:** 10.3390/ijerph19063267

**Published:** 2022-03-10

**Authors:** Eun-Jung Yang, Byung-Sun Choi, Yun-Jung Yang

**Affiliations:** 1 Department of Plastic and Reconstructive Surgery, Yonsei University College of Medicine, Seoul 03722, Korea; enyang7@yuhs.ac; 2 Department of Preventive Medicine, College of Medicine, Chung-Ang University, Seoul 06974, Korea; bschoi@cau.ac.kr; 3 Department of Convergence Science, College of Medicine, Catholic Kwandong University International St. Mary’s Hospital, Incheon 22711, Korea

**Keywords:** phthalates, nonalcoholic liver disease, subclinical hypothyroidism, Korean National Environmental Health Survey

## Abstract

Nonalcoholic fatty liver disease (NAFLD) is a condition of excess accumulation of fats in the liver. Thyroid dysfunction is commonly observed in adult populations with NAFLD. In subjects with thyroid dysfunction, phthalates, which are chemical compounds widely used to increase the flexibility of various plastic products, may increase the risk of NAFLD prevalence. Therefore, our study aimed to evaluate the relationship between the levels of urinary phthalate metabolites and the risk of NAFLD stratified by the levels of thyroid-stimulating hormone (TSH). Data (*n* = 2308) were obtained from the Korean National Environmental Health Survey II (2012–2014). Using the hepatic steatosis index, participants were classified into non-NAFLD (<30) and NAFLD (>36) groups. Participants with euthyroidism were defined as 0.45–4.5 mIU/L for serum TSH and normal thyroxine (T4) levels (*n* = 2125). Subclinical hypothyroidism (SCH) was defined as a higher TSH level (4.5–10 mIU/L) with normal total T4 levels in the serum (*n* = 183). A multivariate analysis was performed to assess the association of the urinary phthalate concentration with the risk of NAFLD after stratification based on the thyroid hormone levels. The levels of phthalate metabolites in urine were not significantly associated with NAFLD in adults with euthyroidism. However, a significant increased risk of NAFLD in those with SCH was observed in the fourth quartile of mono (2-ethyl-5-hydroxyhexyl) phthalate (odds ratio (OR) 13.59, 95% confidence interval (CI) 12.13–86.44), mono (2-ethyl-5-oxohexyl) phthalate (OR 8.55, 95% CI 1.20–60.53), mono-(2-ethyl-5-carboxypentyl) phthalate (OR 9.06, 95% CI 1.78–45.96), and mono-benzyl phthalate (OR 6.05, 95% CI 1.62–22.54) compared to those of the lowest quartile after being adjusted with covariates. In conclusion, the levels of phthalate metabolites in urine are positively associated with NAFLD in adults with SCH. More experimental studies are needed to clarify the risk of NAFLD caused by phthalate exposure in cases with poor thyroid function.

## 1. Introduction

Phthalates are widely used in consumer goods and various personal care products. High-molecular-weight phthalates, including di (2-ethylhexyl) phthalate (DEHP), di-isononyl phthalate, and di-n-octyl phthalate, are mainly used as plasticizers to increase the flexibility of consumer products. Low-molecular-weight phthalates, including diethyl phthalate and di-n-butyl phthalate (DBP), are also used as plasticizers and solvents in cosmetics and pharmaceutical materials [1]. Following entry into the body, these compounds are rapidly metabolized in the liver and gut [2,3,4] and excreted via urine and feces [5,6]. Despite relatively short half-lives, the association of these compounds with adverse health effects is being studied, especially since humans are being continuously exposed to various types of phthalates at the same time. 

Nonalcoholic fatty liver disease (NAFLD) is a condition characterized by marked fat accumulation (≥5%) in the liver without excessive alcohol consumption, viral hepatitis, and other hepatic diseases [7]. The clinical entities associated with this condition range from simple hepatic steatosis to steatohepatitis, liver cirrhosis, and even hepatocellular carcinoma [7]. The global prevalence of NAFLD has increased during the past decades and is estimated to currently be from 20% to 30% [8,9]. A number of diseases, including cardiovascular disease, type 2 diabetes mellitus, chronic kidney disease, and cancer, are associated with the increasing incidence of NAFLD [10,11,12]. Recent epidemiological studies have shown an association between phthalates and various types of liver damage, including NAFLD [13,14,15,16]. 

In addition, thyroid hormones triiodothyronine (T3) and thyroxine (T4) are released from the thyroid gland and have an essential role in growth, development, and energy homeostasis [17]. They are regulated by thyroid-stimulating hormone (TSH) in the pituitary gland. Hypothyroidism is classified as subclinical hypothyroidism (SCH) and overt hypothyroidism. SCH is a condition when the elevated levels of serum TSH are more than the upper reference limit and normal free T4 [18]. Since thyroid hormones are involved in glucose and lipid metabolism [19], poor thyroid function might increase the risk of NAFLD [20,21]. However, there have also been reports of no association between thyroid function and NAFLD [22,23]. Thus, the contribution of thyroid dysfunction in the development of NAFLD is still under discussion. 

The upregulation of the genes involved in cholesterol and fatty acid metabolism in the liver, after interacting with phthalates, has been described in animal studies and in studies on hepatocyte cell lines as a possible underlying mechanism in the development of NAFLD [24,25,26,27,28,29,30]. Additionally, phthalates can disrupt the production of thyroid hormones through binding with thyroid hormone receptors [31] and through downregulation of the thyroid hormone-binding transport protein expression in rat liver [32]. It seems that individuals with thyroid dysfunction may be at an increased risk of NAFLD occurrence if they also have high phthalate concentrations.

Therefore, this study examined the association between the urinary phthalate metabolite levels and NAFLD prevalence in adults with SCH.

## 2. Materials and Methods

### 2.1. Study Population 

This study used the data from the second round of the Korean National Environmental Health Survey (KoNEHS, 2012–2014). KoNEHS has been conducted every 3 years since 2009 to collect nationally representative data on the exposure level of environmental chemicals, the influential factors associated with the exposure, and the spatiotemporal distribution characteristics in the Korean population. For this purpose, KoNEHS II used a two-stage stratified sampling design. Using the 2010 Population and Housing Census of the National Statistical Office as the target population, the first stratification was performed in regional administrative districts and coastal floors, and the second stratification was carried out on a proportion of apartments closely related in socioeconomic level, as well as a proportion of people who engage in agriculture and fishing. Finally, 358 general survey areas were extracted, and a total of 400 sample survey areas, including 42 designated survey areas, were selected; sample households within the survey area were systematically extracted, and about 15 people per sample area were investigated [33].

**Figure 1:**
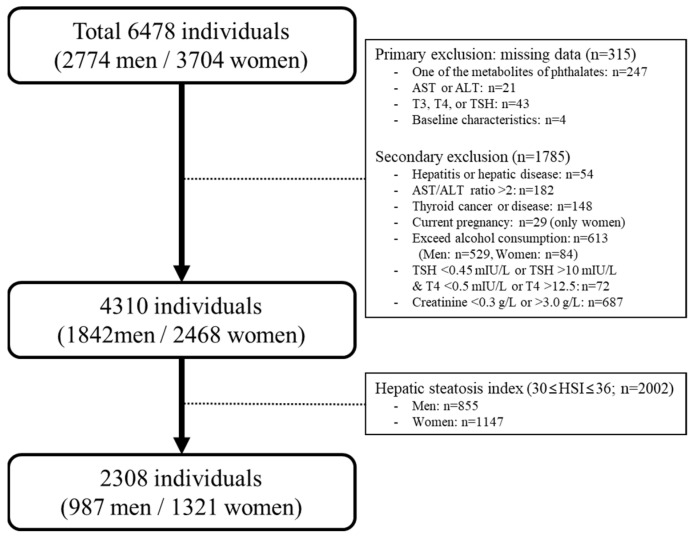
Population in the present study obtained from the second round of the Korean National Environmental Health Survey II (2012–2014).

A total of 6478 participants (2774 men and 3704 women) aged ≥19 years were included. A questionnaire survey, physical measurements, and collection of biological samples (blood and urine) were conducted for each participant. The survey was conducted through a face-to-face interview method, and the collection and management of the biological samples was performed according to the manuals. Among the participants, 315 participants with missing data were excluded: 247 with missing values for phthalate metabolites, 21 with missing data on aspartate transaminase (AST) or alanine aminotransferase (ALT) levels, 43 with missing values for thyroid hormone concentrations, and 4 with missing baseline characteristics. In addition, those with hepatitis or hepatic disease (*n* = 54), AST/ALT ratio > 2 (*n* = 182), thyroid cancer or thyroid disease (*n* = 148), current pregnancy (*n* = 29, only women), excessive alcohol consumption (*n* = 613; 529 men and 84 women), abnormal TSH levels (>10 mIU/L or ≤0.45 mIU/L; *n* = 72), and abnormal creatinine levels (>3.0 g/L or <0.3 g/L; *n* = 687) were excluded. Then, 2002 participants (855 men and 1147 women) were excluded based on the hepatic steatosis index score (30 ≤ HIS ≤ 36) to reduce the confounding influence of NAFLD prevalence. Finally, 2308 participants (987 men and 1321 women) were included in this study (Figure 1).

### 2.2. Data Collection and Diagnosis

Demographic and lifestyle characteristics, including age, gender, drinking and smoking status, physical activity, socioeconomic status, education level, marriage, and medications consumed, were surveyed through face-to-face interviews. Based on the responses, some variables were categorized as follows: education (<high school graduate, high school graduate, and ≥ college/university graduate); drinking and smoking status (never, past, and current); physical activity levels (no, moderate, and vigorous); monthly household income (≤1.5, 1.5–3, 3–4.5, and >4.5 million Korean won); and marital status (single, married, and divorced/separated). Excess alcohol consumption was defined as follows: men who consumed 3 or more times per week and 7–9 cups per time and women who consumed 3 or more times per week and 5–6 cups per time [34]. Body mass index (BMI) was calculated as the body weight (kg) divided by height squared (m^2^).

Participants with diagnoses of hepatitis or hepatic steatosis who were presently undergoing treatment or taking drugs were regarded as patients with hepatic disease. Hypertension was defined by a self-reported history of hypertension or of current antihypertensive medication use. Diabetes mellitus (DM) was defined by a self-reported history of DM or of current antidiabetic medication use. Hyperlipidemia was defined as a self-reported history of hyperlipidemia, antihyperlipidemic medication use, high-density lipoprotein cholesterol level ≤ 40 mg/dL, triglyceride level (TG) ≥ 240 mg/dL, or total cholesterol ≥ 200 mg/dL. 

### 2.3. Measurement of Phthalate Metabolites in Urine 

Spot urine samples were collected from participants at the same time as the blood samples were taken. The collected urine samples were immediately stored at 0–4 °C and were subsequently frozen at −20 °C. The levels of phthalate metabolite, including mono (2-ethyl-5-hydroxyhexyl) phthalate (MEHHP), mono (2-ethyl-5-oxohexyl)phthalate (MEOHP), mono (2-ethyl-5-carboxypentyl)phthalate (MECPP), monobenzyl phthalate (MBzP), and mono-n-butyl phthalate (MnBP), were measured using ultra-performance liquid chromatography-mass spectrometry (Xevo TQ-S, Waters, Milford, MA, USA) in urine samples [35]. Values below the detection limits were imputed using the LOD divided by the square root of 2. The limits for detection of MEHHP, MEOHP, MECPP, MBzP, and MnBP were 0.28, 0.26, 0.34, 0.44, and 0.27 ug/L, respectively. 

### 2.4. Definition of Nonalcoholic Fatty Liver Disease

NAFLD was diagnosed using the hepatic steatosis index (HSI) score, because it is considered an effective and noninvasive NAFLD detection marker [36]. HSI is calculated as follows: HSI = 8 × ([ALT]/[AST] ratio) + BMI (+2, if female; +2, if DM). Values of HSI < 30.0 were defined as participants without hepatic steatosis (non-NAFLD), while values of HSI > 36 were defined as indicative of NAFLD [36]. To increase the sensitivity and specificity for the detection of NAFLD, the participants with 30 ≤ HSI ≤ 36 were eliminated.

### 2.5. Definition of Subclinical Hypothyroidism

KoNEHS II included the total T3, T4, and TSH in the collected blood samples, which were measured using a high-performance ADVIA Centaur^®^ XP system (Siemens Diagnostics, Tarrytown, NY, USA). Based on the provided thyroid hormone concentrations, participants with euthyroidism were defined as having a blood TSH within the reference range (0.45–4.5 mIU/L) [18]. Subjects with a serum TSH level 4.5–10 mIU/L with a normal T4 level (5.0–12.5 mIU/L) were defined as SCH. Participants with serum TSH higher than 10 mIU/L or lower than 0.45 mIU/L were excluded to reduce the potential interference of thyroid medication or other thyroid-related diseases. Free T4 levels were not available in the second round of the KoNEHS dataset; thus, the total T4 levels were used. 

### 2.6. Statistical Analysis 

Subjects were classified as non-NAFLD and NAFLD based on the HSI score and as having normal thyroid function and as having subclinical hypothyroidism based on the serum TSH and T4 levels. Considering the stratified two-stage cluster sampling design, sample weights were used to correct any imbalances between the survey sample and the population. The weighted average and standard error (continuous variables) and weighted frequency (categorical variables) were provided for each group. The general characteristics were compared using the *t*-test, and an analysis of variance (continuous variable) or χ^2^ test (categorical variables) was performed.

The distributions of the phthalate metabolite concentrations (μg/L) were skewed to the right; thus, the data were log-transformed before the analysis. Geometric means and geometric standard deviations of the urinary phthalate metabolite levels were shown in each group. The urinary phthalate metabolite levels were categorized into quartiles based on the weighted sample distribution. The lowest quartile of each phthalate metabolite concentration was used as a reference, because there was no cut-off value for assessing the adverse effects of the phthalate metabolites. 

The relationship between the urinary phthalate metabolites levels and NAFLD according to the TSH levels was analyzed using a multivariate logistic regression analysis. Demographic factors, including age; gender; drinking; smoking; physical activity; monthly household income; education; marital status; and clinical variables (including hypertension, DM, and hyperlipidemia) were included in the regression model. Since the BMI is used in the HSI formula, it was not included in the multivariate analysis as an independent variable to avoid collinearity. In this formula, 2 points were added for the BMIs of female participants to account for the lower BMI of women compared to that of men. Further, 2 points were added for the BMIs of subjects with DM, because DM is an independent risk factor of NAFLD [36]. Thus, gender and DM were used as independent variables in the multivariate analysis. Data analyses were performed using STATA (version 16.0, StataCorp LP, College Station, TX, USA). *p*-values <0.05 were considered significant.

## 3. Results

### 3.1. General Characteristics of Study Participants

The demographic and clinical characteristics of the participants (*n* = 2308) are represented in Table 1 and Table 2. According to the HSI score, participants with HSI ≤ 30.0 were classified as non-NAFLD (*n* = 1202), while those with HIS > 36.0 were placed in the NAFLD group (*n* = 1106) (Table 1). The mean age and BMI were significantly higher in subjects with NAFLD compared to those without NAFLD (both *p* < 0.001). The weighted frequencies of gender, drinking and smoking status, monthly household income, education, marital status, and comorbidities were statistically different between the two groups (all *p* < 0.05). 

Based on the TSH and T4 levels, participants with euthyroidism and SCH were classified (Table 2). Subjects with 0.45–4.5-mIU/L TSH and normal T4 (5.0–12.5 mIU/L) in the serum were defined as euthyroidism (*n* = 2125). SCH was defined as 4.5–10-mIU/L TSH and normal T4 in the serum (*n* = 183). The average age was significantly higher in participants with SCH compared to those with euthyroidism (*p* = 0.002). The weighted frequencies of gender, drinking and smoking status, education, and marital status were significantly different between the two groups (all *p* < 0.05). However, the BMI, physical activity, monthly household income, and comorbidities in the subjects with euthyroidism were not different compared to the subjects with SCH. 

### 3.2. Prevalence of NAFLD in Participants with Euthyroidism and with SCH 

The weighted prevalence of NAFLD based on HSI was 45.23% (95% CI: 42.37–48.11) in participants with euthyroidism and 50.19% (95% CI: 40.72–59.63) in participants with SCH (Table 3). The prevalence of NAFLD was not different between participants with euthyroidism and those with SCH (*p* = 0.321). 

### 3.3. The Concentrations of Phthalate Metabolites in Urine 

The levels of phthalate metabolites in urine are represented in Table 4. The urinary phthalate metabolite concentrations were not statistically different between those with non-NAFLD and NAFLD and normal thyroid function. 

In the participants with subclinical hypothyroidism, the levels of MEHHP, MEOHP, MECPP, and MBzP in their urine were significantly higher in the NAFLD group when compared to those without NAFLD (*p* = 0.001, *p* = 0.028, *p* = 0.011, and *p* = 0.037, respectively). However, there was no significant difference in the MnBP concentrations in urine between the non-NAFLD and NAFLD groups. 

### 3.4. The Association between Urinary Phthalate Metabolites and NAFLD after Stratified by TSH Levels 

Participants were classified into two groups based on thyroid function: those with euthyroidism and those with SCH, according to the serum TSH and T4 levels (Table 5). Subjects were also divided into non-NAFLD and NAFLD groups based on the HSI score. Univariate and multivariate analyses were conducted to examine the association between urinary phthalate metabolite levels and NAFLD in subjects with normal thyroid function andSCH. 

Subjects with euthyroidism did not display a significant increase in the prevalence of NAFLD when considering the increasing urinary phthalate metabolites levels in both the univariate and multivariate analyses. The urinary MnBP levels were correlated with NAFLD in the results of the trend tests after adjustment for age, gender, creatinine, smoking, drinking, exercise, marital status, education, income, hypertension, diabetes mellitus, and hyperlipidemia (*p* = 0.032). 

In participants with SCH, the risk of NAFLD in the fourth quartile of MEHHP, MECPP, and MBzP was significantly higher than that in those in the lowest quartile in the univariate analysis (OR 3.87 (95% CI 1.40–10.70), OR 3.29 (95% CI 1.19–9.08), and OR 3.23 (95% CI 1.08–9.68), respectively). After adjustment for demographic and clinical factors, participants in the fourth quartile of MEHHP, MEOHP, MECPP, and MBzP displayed a significant increase of NAFLD risk when compared to those in the lowest quartile (OR 13.59 (95% CI 2.13–86.44), OR 8.55 (95% CI 1.20–60.53), OR 9.06 (95% CI 1.78–45.96), and OR 6.05 (95% CI 1.62–22.54), respectively). The results of the trend tests for the correlation of MEHHP, MEOHP, MECPP, and MBzP with NAFLD were *p* < 0.05 in both the crude analysis and multivariate analysis.

## 4. Discussion

In this study, we aimed to assess the relationship between the urinary phthalate metabolites levels and NAFLD in subjects with SCH. Based on the serum TSH and T4 levels, participants were classified into euthyroidism and SCH. There was no significant relationship between thyroid function and NAFLD. A significant relationship between the urinary phthalate metabolite levels and NAFLD prevalence was not observed in participants with euthyroidism both the univariate and multivariate analyses. However, in subjects with SCH, the highest quartile of MEHHP, MEOHP, MECPP, and MBzP in urine displayed a significant association with NAFLD prevalence after adjusting for demographic and clinical factors. 

Phthalates are widely used to increase flexibility in consumer products. They can enter the human body through ingestion, inhalation, and dermal routes [37]. The phthalates are rapidly hydrolyzed to monoesters in the liver and gut [2,3,4], and the monoesters of DEHP are additionally metabolized into secondary oxidized metabolites such as MEHHP, MEOHP, and MECPP [3,5]. Most of the metabolites are excreted through the urine and feces within 24 h [5,6]. Thus, urine samples are commonly used to identify phthalate exposure levels. 

A Westernized diet and sedentary lifestyle might be considered important risk prerequisite factors in the development of NAFLD [38,39]. Despite cross-sectional studies that have reported the association between NAFLD and liver damage with urinary phthalate metabolite levels in the general population [13,14], clarification of the relationship between phthalates and NAFLD pathogenesis is needed. Experimental studies have described the underlying mechanism in the pathogenesis of NAFLD after phthalate exposure. DEHP treated with high fat or oleic acid increased the hepatic TG levels and increased the gene and protein expressions of the key enzymes involved in fatty acid synthesis through the induction of nuclear receptors such as peroxisome proliferator-activated receptors (PPARs) and sterol regulatory element-binding protein 1 (SREBP-1c) in rats [24,29] and the HepG2 cell line [25]. In addition, the disruption of genes associated with the liver, such as those involved in fatty acid metabolism and the development of NAFLD, was identified after exposure to environmentally relevant levels of phthalates in zebrafish [26]. When HepG2 cells were treated with MEHP, the metabolite of DEHP, lipid accumulation was increased through the disruption of several key enzymes. This was linked to the de novo synthesis of fatty acids [28]. Perinatal exposure to DEHP also increased the TG levels, as well as the overexpression of diacylglycerol O-acyltransferase 1 (DGAT1), which is involved in re-esterification from free fatty acid to TG in the liver of adult male offspring [40]. Accordingly, phthalates could induce the development of NAFLD through disrupting the expression of lipogenic enzymes. 

In addition, phthalates were found to influence not only increased liver fat mass but, also, thyroid hormones homeostasis [41,42]. Thyroid hormones are released from the thyroid gland and regulated by TSH in the pituitary gland. The levels of phthalate metabolites showed a negative association with the serum total T4 and free T4 levels in pregnant women [43] and with the serum-free T4 and T3 levels in men [44]. The urinary phthalate concentrations were shown to be associated with the alteration of thyroid hormones in the Korean adult population [45]. Epidemiologic studies have demonstrated that FRTL-5 rat thyroid follicular cells treated with phthalates showed increased iodide uptake [46]. Exposure to DBP increased the serum T3 levels and decreased the serum TSH levels in hyperthyroid rats compared to the control animals [47]. Additionally, DEHP significantly decreased the serum T3 and T4 levels, as well as the thyrotropin-releasing hormone levels, in rats [32,41]. Based on these epidemiologic and laboratory studies, phthalates may induce the disruption of thyroid function.

The disruption of thyroid hormones may contribute to NAFLD pathogenesis [20,21]. When the circulating thyroid hormone levels were reduced, resting energy expenditure, weight gain, and lipolysis were decreased, and cholesterol levels were increased [48]. In a previous meta-analysis, subjects with hypothyroidism were 52% more likely to develop NAFLD compared to euthyroid subjects [49]. Thyroid hormones may modulate the metabolic process through the regulation of nuclear receptors, including PPARs, liver X receptor, and others [50]. Since the mechanism of phthalate interference with hepatic lipid metabolism involves their direct or indirect activity on nuclear receptors [24,25,29], subjects with diminished thyroid action might have an increased risk of NAFLD when exposed to phthalates compared to subjects with normal thyroid function. 

To the best of our knowledge, this is the first study to report the association between urinary phthalate metabolite levels and NAFLD in subjects with subclinical hypothyroidism. However, some limitations of the study need to be addressed. First, subjects with NAFLD were diagnosed using the HSI score instead of a liver biopsy or imaging modalities, such as ultrasonography and computed tomography. Despite a liver biopsy being the gold standard for assessing the severity of hepatic steatosis, it does not appear to be cost-effective for mass screening. HSI is a noninvasive tool that is able to predict the presence of NAFLD. In the Korean population, an area under receiver operating curve of the HSI was 0.812 (95% CI 0.801–0.824) [36]. At a value of <30.0, the HSI could exclude NAFLD with a sensitivity of 92.5% (95% CI 91.4–93.5), and at a value of >36.0, the HSI could detect NAFLD with a specificity of 92.4% (95% CI 91.3–93.4). Thus, we ruled out the group with intermediate values (30 ≤ HSI ≤ 36) to increase the accuracy of the HSI for predicting NAFLD. Second, individuals with TSH levels higher than the upper reference limit and with a normal free T4 level were diagnosed with SCH [51]. However, free T4 levels were not available in the second round of the KoNEHS dataset. Thus, subclinical hypothyroidism was defined in subjects using serum TSH levels ranging from 4.5 mIU/L to 10 mIU/L and normal T4 levels. Even in the case of asymptomatic subclinical hypothyroidism, medications could be prescribed that may cause TSH to exceed 10 mIU/L or be lower than 0.45 mIU/L in the serum; thus, both these values were excluded from the analysis. Third, the second round of KoNEHS data set did not provide data on the amount of alcohol consumption (g/day). We defined heavy drinkers as those who consumed alcohol more than 3 times a week and consumed 7–9 glasses per sitting in men (5–6 glasses per sitting in women) [34]. Fourth, phthalate metabolites were measured using single spot urine samples. Since phthalates have relatively short half-lives, intra- and inter-variations might be present. However, KoNEHS randomly collected a sufficient number of spot urine samples. Thus, it may adequately reflect the average phthalate metabolite levels in the population. Finally, because the number of participants with subclinical hypothyroidism was relatively small (*n* = 183), it is difficult to generalize these results to the larger population. Further studies are needed to clarify whether phthalates are independently associated with NAFLD pathogenesis in subjects with subclinical and overt thyroid dysfunction. 

## 5. Conclusions

Our study suggests an association between phthalates and the risk of NAFLD in subjects with subclinical hypothyroidism. However, in participants with normal thyroid function, a positive relationship was not clearly observed. Therefore, it is desirable to reduce phthalate exposure in order to prevent NAFLD, especially in individuals with poor thyroid function. In addition, the molecular mechanism of increased NAFLD risk caused by phthalates in the case of poor thyroid function should be assessed. 

## Figures and Tables

**Table 1:** General characteristics of the study participants based on the hepatic steatosis index.

	Total (*n* = 2308)	Non-NAFLD (*n* = 1202)	NAFLD (*n* = 1106)	*p*-Value
Age, y	43.98 ± 0.53	41.60 ± 0.65	46.81 ± 0.69	<0.001
Gender (men, %)	49.23 ± 1.33	45.14 ± 1.91	54.12 ± 1.93	0.002
BMI (kg/m^2^)	24.19 ± 13.08	20.81 ± 0.07	28.23 ± 0.11	<0.001
Drinking Status (%)				0.016
Never	31.60 ± 1.22	28.51 ± 1.50	35.29 ± 2.01	
Former	4.04 ± 0.53	4.54 ± 0.80	3.45 ± 0.65	
Current	64.35 ± 1.27	66.94 ± 1.63	61.24 ± 2.10	
Smoking Status (%)				0.002
Never	64.76 ± 1.41	67.95 ± 1.84	60.95 ± 1.96	
Former	13.70 ± 0.92	13.84 ± 1.27	13.53 ± 1.31	
Current	21.53 ± 1.26	18.20 ± 1.56	25.50 ± 1.90	
Physical activity (%)				0.280
No	64.30 ± 1.40	62.72 ± 1.97	66.19 ± 1.93	
Moderate	21.05 ± 1.13	22.23 ± 1.56	19.65 ± 1.67	
Vigorous	14.63 ± 1.01	15.04 ± 1.41	14.15 ± 1.30	
Monthly household income (%)				0.001
≤1.5 million KRW	21.03 ± 1.58	17.62 ± 1.63	25.10 ± 2.13	
1.5–3 million KRW	34.54 ± 1.66	34.81 ± 2.07	34.22 ± 2.19	
3–4.5 million KRW	18.67 ± 1.33	19.23 ± 1.75	18.00 ± 1.72	
>4.5 million KRW	25.74 ± 1.86	28.33 ± 2.25	22.66 ± 2.21	
Education (%)				<0.001
<Middle school	20.37 ± 1.26	16.05 ± 1.32	25.53 ± 1.83	
Middle-High school	40.65 ± 1.53	41.63 ± 1.96	39.48 ± 2.10	
≥College/University	38.97 ± 1.68	42.31 ± 2.16	34.97 ± 2.20	
Marital status (%)				<0.001
Single	23.82 ± 1.55	30.06 ± 2.14	16.36 ± 1.84	
Married	68.07 ± 1.65	63.12 ± 2.23	73.99 ± 2.04	
Divorced/Separated	8.09 ± 0.80	6.80 ± 0.89	9.64 ± 1.19	
Comorbidities				
Hypertension	14.19 ± 0.94	6.74 ± 0.77	23.10 ± 1.65	<0.001
Hyperlipidemia	7.46 ± 0.67	1.32 ± 0.28	14.81 ± 1.35	<0.001
Diabetes mellitus	30.82 ± 1.16	15.22 ± 1.27	49.47 ± 1.87	<0.001

Data was presented as the weighted mean or frequency ± standard errors, as appropriate. NAFLD: nonalcoholic fatty liver disease, BMI: body mass index, KRW: Korean won.

**Table 2:** General characteristics of the study participants based on the thyroid function.

	Total (*n* = 2308)	Euthyroidism (*n* = 2125)	Subclinical Hypothyroidism (*n* = 183)	*p*-Value
Age, y	43.98 ± 0.53	43.59 ± 0.54	49.36 ± 1.84	0.002
Gender (men, %)	49.23 ± 1.33	50.04 ± 1.37	31.58 ± 4.80	<0.001
BMI (kg/m^2^)	24.19 ± 13.08	24.16 ± 0.13	24.64 ± 0.40	0.240
Drinking Status (%)				0.009
Never	31.60 ± 1.22	30.74 ± 1.27	43.65 ± 4.92	
Former	4.04 ± 0.53	4.06 ± 0.56	3.77 ± 1.99	
Current	64.35 ± 1.27	65.18 ± 1.31	52.56 ± 4.68	
Smoking Status (%)				<0.001
Never	67.76 ± 1.41	63.33 ± 1.48	84.84 ± 3.07	
Former	13.70 ± 0.92	13.80 ± 0.97	12.30 ± 2.91	
Current	21.53 ± 1.26	22.85 ± 1.34	2.85 ± 1.23	
Physical activity (%)				0.540
No	64.30 ± 1.40	64.47 ± 1.47	61.88 ± 4.55	
Moderate	21.05 ± 1.13	21.00 ± 1.18	21.74 ± 4.20	
Vigorous	14.63 ± 1.01	14.51 ± 1.05	16.36 ± 3.39	
Monthly household income (%)				0.253
≤1.5 million KRW	21.03 ± 1.58	20.73 ± 1.60	25.28 ± 3.96	
1.5–3 million KRW	34.54 ± 1.66	34.47 ± 1.69	35.48 ± 4.65	
3–4.5 million KRW	18.67 ± 1.33	18.91 ± 1.36	15.20 ± 3.69	
>4.5 million KRW	25.74 ± 1.86	25.87 ± 1.90	24.01 ± 4.35	
Education (%)				0.041
<Middle school	20.37 ± 1.26	19.77 ± 1.29	28.75 ± 4.40	
Middle-High school	40.65 ± 1.53	40.71 ± 1.60	39.77 ± 4.67	
≥College/University	38.97 ± 1.68	39.50 ± 1.72	31.47 ± 5.05	
Marital status (%)				0.038
Single	23.82 ± 1.55	24.55 ± 1.56	13.51 ± 5.05	
Married	68.07 ± 1.65	67.46 ± 1.66	76.65 ± 5.23	
Divorced/Separated	8.09 ± 0.80	7.97 ± 0.82	9.83 ± 2.61	
Comorbidities				
Hypertension	14.19 ± 0.94	13.99 ± 0.99	17.12 ± 3.33	0.372
Hyperlipidemia	7.46 ± 0.67	30.57 ± 1.20	34.45 ± 4.65	0.422
Diabetes mellitus	30.82 ± 1.16	7.18 ± 0.67	11.49 ± 2.80	0.121

Data was presented as the weighted mean or frequency ± standard errors, as appropriate. BMI: body mass index, KRW: Korean won.

**Table 3:** Prevalence of NAFLD in participants with euthyroidism and with SCH.

		Euthyroidism (*n* = 2125)	Subclinical Hypothyroidism (*n* = 183)	*p*-Value
NAFLD	Actual number	1007	99	0.321
	Weighted frequency (95% CI)	45.23 (42.37–48.11)	50.19 (40.72–59.63)	

NAFLD: nonalcoholic fatty liver disease, SCH: Subclinical hypothyroidism, CI: confidence interval.

**Table 4:** The average of the urinary phthalate metabolite levels (log transformed, μg/L) in the study population.

	Concentrations (GM [95% CI])			
	Total (*n* = 2308)	Non-NAFLD (*n* = 1202)	NAFLD (*n* = 1106)	*p*-Value
Euthyroidism (*n* = 2125)				
MEHHP	3.02 (2.97–3.06)	2.99 (2.92–3.05)	3.05 (2.98–3.12)	0.070
MEOHP	2.66 (2.61–2.71)	2.67 (2.61–2.74)	2.65 (2.58–2.72)	0.957
MECPP	3.15 (3.10–3.19)	3.14 (3.08–3.20)	3.16 (3.10–3.22)	0.353
MnBP	3.32 (3.26–3.39)	3.36 (3.28–3.43)	3.28 (3.20–3.37)	0.210
MBzP	1.13 (1.05–1.21)	1.11 (1.02–1.20)	1.15 (1.04–1.26)	0.209
Subclinical hypothyroidism (*n* = 183)				
MEHHP	3.10 (2.98–3.23)	2.93 (2.74–3.11)	3.28 (3.11–3.44)	0.001
MEOHP	2.76 (2.64–2.89)	2.62 (2.44–2.80)	2.91 (2.72–3.09)	0.028
MECPP	3.22 (3.11–3.33)	3.11 (2.94–3.27)	3.33 (3.18–3.49)	0.011
MnBP	3.35 (3.22–3.48)	3.35 (3.17–3.53)	3.35 (3.18–3.52)	0.971
MBzP	1.02 (0.84–1.20)	0.85 (0.59–1.12)	1.18 (0.95–1.40)	0.037

GM: geometric mean, CI: confidence interval, NAFLD: nonalcoholic fatty liver disease, SCH: subclinical hypothyroidism, MEHHP: mono (2-ehtyl-5-hydroxyhexyl) phthalate, MEOHP: mono (2-ethyl-5-oxohexyl) phthalate, MECPP: mono (2-ethyl-5-carboxypentyl) phthalate, MnBP: mono-n-butyl phthalate, MBzP: mono-benzyl phthalate. Euthyroidism was defined as 0.45–4.5-mIU/L TSH and normal T4 (5.0–12.5 mIU/L) in the serum. Subclinical hypothyroidism was defined as 4.5–10-mIU/L TSH and normal T4 in the serum.

**Table 5:** The association between urinary phthalate metabolites and NAFLD after being classified into participants with euthyroidism and with SCH.

	**Euthyroidism (*n* = 2125)**				**Subclinical Hypothyroidism (*n* = 183)**			
	Crude Analysis		Multivariate Analysis		Crude Analysis		Multivariate Analysis	
	OR (95% CI)	*p* for Trend	OR (95% CI)	*p* for Trend	OR (95% CI)	*p* for Trend	OR (95% CI)	*p* for Trend
MEHHP								
Quartile 1	1	0.072	1	0.841	1	0.003	1	0.006
Quartile 2	1.19 (0.87–1.64)		1.09 (0.74–1.06)		1.00 (0.34–2.96)		2.18 (0.59–8.02)	
Quartile 3	1.37 (1.02–1.85)		1.21 (0.81–1.80)		2.78 (0.90–8.52)		3.22 (0.68–15.13)	
Quartile 4	1.31 (0.93–1.83)		1.01 (0.65–1.56)		3.87 (1.40–10.70)		13.59 (2.13–86.44)	
MEOHP								
Quartile 1	1	0.956	1	0.097	1	0.038	1	0.033
Quartile 2	0.94 (0.69–1.29)		0.77 (0.53–1.10)		0.89 (0.28–2.85)		1.46 (0.34–6.20)	
Quartile 3	1.08 (0.78–1.49)		0.76 (0.50–1.16)		1.90 (0.63–5.72)		1.72 (0.41–7.15)	
Quartile 4	0.96 (0.69–1.34)		0.66 (0.41–1.04)		2.55 (0.91–7.10)		8.55 (1.20–60.53)	
MECPP								
Quartile 1	1	0.360	1	0.421	1	0.020	1	0.024
Quartile 2	0.94 (0.69–1.27)		0.72 (0.51–1.01)		1.18 (0.36–3.80)		2.57 (0.76–8.60)	
Quartile 3	1.22 (0.91–1.62)		0.86 (0.58–1.29)		1.76 (0.56–5.54)		1.38 (0.30–6.22)	
Quartile 4	1.07 (0.77–1.49)		0.77 (0.50–1.19)		3.29 (1.19–9.08)		9.06 (1.78–45.96)	
MnBP								
Quartile 1	1	0.209	1	0.032	1	0.914	1	0.930
Quartile 2	0.98 (0.71–1.36)		0.86 (0.59–1.26)		1.78 (0.58–5.48)		1.01 (0.25–4.11)	
Quartile 3	0.85 (0.62–1.15)		0.64 (0.40–1.00)		0.75 (0.25–2.28)		0.68 (0.16–2.88)	
Quartile 4	0.84 (0.60–1.16)		0.60 (0.35–1.01)		1.41 (0.48–4.09)		1.06 (0.22–5.08)	
MBzP								
Quartile 1	1	0.210	1	0.840	1	0.043	1	0.009
Quartile 2	1.19 (0.87–1.64)		1.15 (0.79–1.67)		1.97 (0.68–5.67)		2.37 (0.58–9.55)	
Quartile 3	1.14 (0.84–1.56)		1.14 (0.77–1.69)		1.84 (0.61–5.51)		2.14 (0.66–6.90)	
Quartile 4	1.23 (0.92–1.63)		1.04 (0.70–1.56)		3.23 (1.08–9.68)		6.05 (1.62–22.54)	

OR: Odds ratio; CI: Confidence interval; Q: Quartile; MEHHP: mono (2-ehtyl-5-hydroxyhexyl) phthalate; MEOHP: mono (2-ethyl-5-oxohexyl) phthalate; MECPP: mono (2-ethyl-5-carboxypentyl) phthalate; MnBP: mono-n-butyl phthalate; MBzP: mono-benzyl phthalate. Multivariate model was adjusted for age, gender, creatinine, smoking, drinking, exercise, marital status, education, income, hypertension, diabetes mellitus, and hyperlipidemia. Normal thyroid function was defined as 0.45–4.5-mIU/L serum thyroid-stimulating hormone (TSH) and normal serum thyroxine (T4) (5.0–12.5 mIU/L). Subclinical hypothyroidism was defined as 4.5–10-mIU/L serum TSH and normal serum T4.

## Data Availability

This study used data from the Second Korean National Environmental Health Survey (KoNEHS), which was conducted by the Ministry of Environment, National Institute of Environmental Research. The data presented in this study are available on request from the corresponding author. The data are not publicly available due to protected personal information.
